# Effect of mind mapping combined with the SBAR shift turnover model on reducing nursing defects during bedside shift turnovers after cardiac stenting in cardiology

**DOI:** 10.3389/fsurg.2025.1666274

**Published:** 2026-01-12

**Authors:** Sujuan Che, Fangfang Chen, Jingjing Liu, Jie Wang, Rongrong Miao

**Affiliations:** Department of Cardiology, Taihe County People’s Affiliated Taihe Hospital of Wannan Medical College, Fuyang, Anhui, China

**Keywords:** cardiology, mind map, nursing defects, SBAR, shift turnover

## Abstract

**Aim:**

This study aims to explore the effect of a mind mapping technique combined with the situation–background–assessment–recommendation (SBAR) shift turnover model on reducing nursing defects during bedside shift turnovers after cardiac stenting in cardiology.

**Methods:**

This quasi-experimental study retrospectively analyzed clinical data of 96 patients who received cardiac stenting at our hospital between August 2022 and August 2023. According to the time of admission, patients were divided into an observation group (OG, *n* = 48) and a control group (CG, *n* = 48). Fourteen nurses participated in the study; they were matched by professional level and then randomly assigned to the OG and CG (*n* = 7 per group). The CG adopted the traditional mode of shift turnover. Based on the CG approach, the OG adopted a mind map combined with the SBAR shift turnover model. Outcomes compared between the two groups included the quality of shift turnover, nurses’ mastery of patient condition, handover time, defect rate of shift turnover, incidence of nursing adverse events and complaints, and patient satisfaction.

**Results:**

Compared with the CG, the OG showed higher scores for shift turnover quality and nurses’ mastery of patient conditions, shorter handover time, and higher patient satisfaction (*P* < 0.05). The defect rate of shift turnover and the incidence of nursing adverse events and complaints were lower in the OG; however, these differences were not statistically significant.

**Conclusion:**

In this single-center study, integrating a mind map-supported SBAR handover into bedside shift handovers was associated with shorter handover time, fewer observed handover defects, and higher patient satisfaction. These findings suggest potential clinical benefits for communication safety after cardiac stenting; however, the results should be interpreted cautiously due to the small nurse-level sample size and study design.

## Introduction

Cardiac stenting is commonly used in the clinical treatment of coronary atherosclerotic heart disease (coronary heart disease) and other cardiovascular diseases. By dilating the diseased coronary artery, it restores myocardial perfusion, thereby improving cardiac blood supply and maintaining normal cardiac function (1). However, due to surgical risks and numerous postoperative precautions, the lack of standardized nursing care can not only increase patient pain due to complications but also negatively affect postoperative recovery and prognosis (2).

The nurse shift system is a core component of hospital management, ensuring continuity of clinical nursing care and reducing the incidence of medical and nursing disputes (3). At present, traditional nurse shift handovers are usually conducted either orally in groups or at the bedside, with night shifts often relying on group oral reports (4). This approach is often mechanical and repetitive, consisting mainly of reading shift reports without logical organization, and bedside handovers sometimes become a mere formality (5). Therefore, intervening effectively in the shift handover process is essential to improving the overall quality of nursing communication and patient care.

Mind mapping is a cognitive organizational tool developed in the early 1970s by Tony Buzan, a renowned British psychologist and educator. It uses words and images to integrate visual and logical thinking, representing ideas through thematic keywords and imagery. By creating a dendritic structure, mind maps connect themes across multiple levels, thereby improving creativity and memory (6). In recent years, mind mapping has received increasing attention in China and has been widely applied in nursing education and clinical practice (7). According to related studies, the situation–background–assessment–recommendation (SBAR) standardized communication model has been widely adopted in the medical systems across many European and American countries (8). As an effective handover communication tool, SBAR facilitates information exchange among healthcare providers and plays an important role in ensuring quality of care and patient safety (9).

Therefore, the present study aimed to explore the effect of combining mind mapping with the SBAR shift turnover model on reducing nursing defects during bedside handovers following cardiac stenting in the cardiology department.

## Data and methods

### General data

Our study was approved by the Medical Ethics Committee of our hospital. Clinical data from 96 patients who received cardiac stenting treatment at our hospital between August 2022 and August 2023 were retrospectively analyzed.

Patients were included if they (1) had a confirmed coronary heart disease; (2) met the indications for cardiac stent implantation; and (3) had complete and authentic clinical data. Patients were excluded if they had (1) concomitant malignant tumors or other major diseases; (2) severe electrolyte disorders; (3) a recent history of bleeding; (4) allergies to iodine-based contrast agents or stent materials; (5) a history of cardiac surgery; (6) severe hepatic, renal, or other parenchymal organ disease or decompensation; or (7) schizophrenia or other mental disorders.

This study employed a quasi-experimental design. According to the time of admission, patients were assigned to the OG (48 cases) or CG (48 cases). The CG included 25 men and 23 women aged 18–68 years (mean age 41.85 ± 4.24 years), while the OG group included 26 men and 22 women aged 20–70 years (mean age 41.96 ± 4.35 years). There were no statistically significant differences between the two groups in terms of gender or age (*P* > 0.05). A total of 14 nurses participated in the study (10 women, four men; age 19–37 years, mean age 26.31 ± 4.22 years). Among them, ten held college or undergraduate degrees and four were secondary school graduates. Nurses were matched according to professional level and randomly assigned to the OG and CG (*n* = 7 per group). No significant differences were observed in baseline characteristics between the two groups (*P* > 0.05).

### Methods

The CG adopted the traditional shift turnover model, in which nurses conducted bedside handovers according to the conventional process, primarily involving reviewing written materials, disease-related information, environmental instruments, equipment and medications, and the results of comprehensive physical examinations.

Based on the CG approach, the OG adopted a mind map combined with the SBAR shift turnover model, as described below:

1. A mind map was specifically developed for patients after cardiac stent implantation. Based on the nursing points and experience of patients after interventional surgery, the mind map used postoperative care as the key word. First-level branches included patient psychological status, stent placement, assessment of blood leakage or hematoma at the puncture site, pain assessment, limb placement, fluid intake, urine output, compliance with medical advice. Patient psychological status was further divided into anxiety, tension, fear, and other secondary branches. Stent placement was subdivided into the condition of the diseased vessels and the location and number of stents implanted. Oozing and hematoma were further divided into two subcategories, such as the area of oozing or hematoma, dressing contamination, and corresponding medical treatment. Pain assessment was subdivided into the nature and duration of pain and whether analgesic drugs were used. Compliance with medical advice was further divided into three branches: average, good, and poor. Each branch was organized into a chart and assembled into a display board, which was clear and concise, enabling responsible nurse to grasp key patient information.
2. Each day after the patient returned to the ward following stent implantation, the responsible nurse assessed the condition of the patient, identified existing and potential nursing risks, and created a corresponding mind map. During bedside handover, guided by the mind map, the nurse conducted the handover using the SBAR framework, covering the current status of the patient, background information, identified problems, and care recommendations.
3. The project leader and head nurse summarized the findings on a weekly basis, identified any deficiencies, and discussed them during the Monday morning meeting to formulate targeted improvement strategies.

#### Observation indicators

1. Shift handover quality: The quality of shift handover in the two groups was evaluated using a self-designed shift quality assessment scale, which consisted of three dimensions: information quality, behavioral norms, and interactive support. Each dimension was scored on a 25-point scale, with higher scores indicating better handover quality.
  Instrument details: The shift handover quality scale comprised 15 items across three domains (information quality, behavioral norms, and interactive support), with five items per domain. Items were rated on a five-point Likert scale (1 = not met at all; 5 = fully met). Domain scores ranged from 5 to 25, and the total scale score from 15 to 75, with higher scores indicating better handover quality. Content validity was established through expert review by senior cardiology nursing leaders, who evaluated item relevance, clarity, and alignment with each domain. Internal consistency reliability in our sample was acceptable for both the total scale and for each domain, with Cronbach's *α* values exceeding 0.80.
2. Mastery of the patient condition: Nurses’ understanding of the patient condition, treatments, health education, and daily care was assessed using the Patient Condition Mastery Scale developed by the geriatrics department. Each item was scored on a 10-point scale, with higher scores indicating better mastery of the patient condition.
3. Handover time: The average duration of nurse handovers in both groups was recorded and compared.
4. Defect rate of shift turnover: The defect rate included cases of incomplete handover content and incomplete handover record forms.
5. Incidence of nursing adverse events and complaints: The frequency of nursing risk events related to handover errors and patient complaints was recorded and compared between the two groups.
6. Nursing satisfaction: Patient satisfaction was assessed using a self-developed nursing satisfaction questionnaire, with a total score of 100 points. Scores of 80–100 indicated “very satisfied,” 60–79 indicated “basically satisfied,” and <60 indicated “dissatisfied.” The total satisfaction rate was calculated as (very satisfied + basically satisfied)/total  ×  100%. The questionnaire was self-developed by the research team with reference to the structure and dimensions of the validated Newcastle Satisfaction with Nursing Scale (10), which has been widely used to assess the quality of nursing care from the patient perspective. Before use, three senior nursing experts reviewed the items to establish content validity, and a pilot test involving 20 patients demonstrated good internal consistency (Cronbach's *α* = 0.87).

Unit of analysis: Outcomes (1)–(5)—shift turnover quality, mastery of the patient condition, handover time, defect rate of shift turnover, and the incidence of nursing adverse events and complaints—were analyzed at the nurse level (*n* = 7 per group). For these outcomes, each nurse's metric represents an aggregation across that nurse's bedside handovers during the study period. Outcome (6), nursing satisfaction, was analyzed at the patient level (*n* = 48 per group).

### Statistical analysis

SPSS 24.0 statistical software was used for data analysis. Nurse-level outcomes (handover quality scores, mastery scores, handover time, defect rate, adverse events, and complaints) were analyzed using the nurse as the unit of analysis (*n* = 7 per group). Patient-level nursing satisfaction was analyzed using the patient as the unit of analysis (*n* = 48 per group). For continuous nurse-level outcomes, distributional assumptions were evaluated prior to hypothesis testing: normality was assessed using the Shapiro–Wilk test and inspection of Q–Q plots, and homogeneity of variances was assessed using Levene's test. When assumptions were satisfied, between-group comparisons were conducted using two-sample Student's *t*-tests (two-sided); when variances were unequal, Welch's *t*-tests were used. If normality assumptions were violated, the Mann–Whitney *U*-test was used, and results were summarized as median (IQR) in addition to mean ± SD. Categorical outcomes were compared using Fisher's exact test (two-sided) for 2 × 2 tables; for tables larger than 2 × 2, the Fisher–Freeman–Halton exact test was used (with Monte Carlo estimation applied when needed). We did not pool patient-level observations with nurse-level observations, and no cross-level tests were performed. Measurement data are expressed as mean ± SD when approximately normally distributed and as median (IQR) when non-normally distributed, while count data are expressed as *n* (%). A two-sided *P* < 0.05 was considered statistically significant.

## Results

### Shift turnover quality of nurses in the two groups (nurse-level)

**Figure 1:**
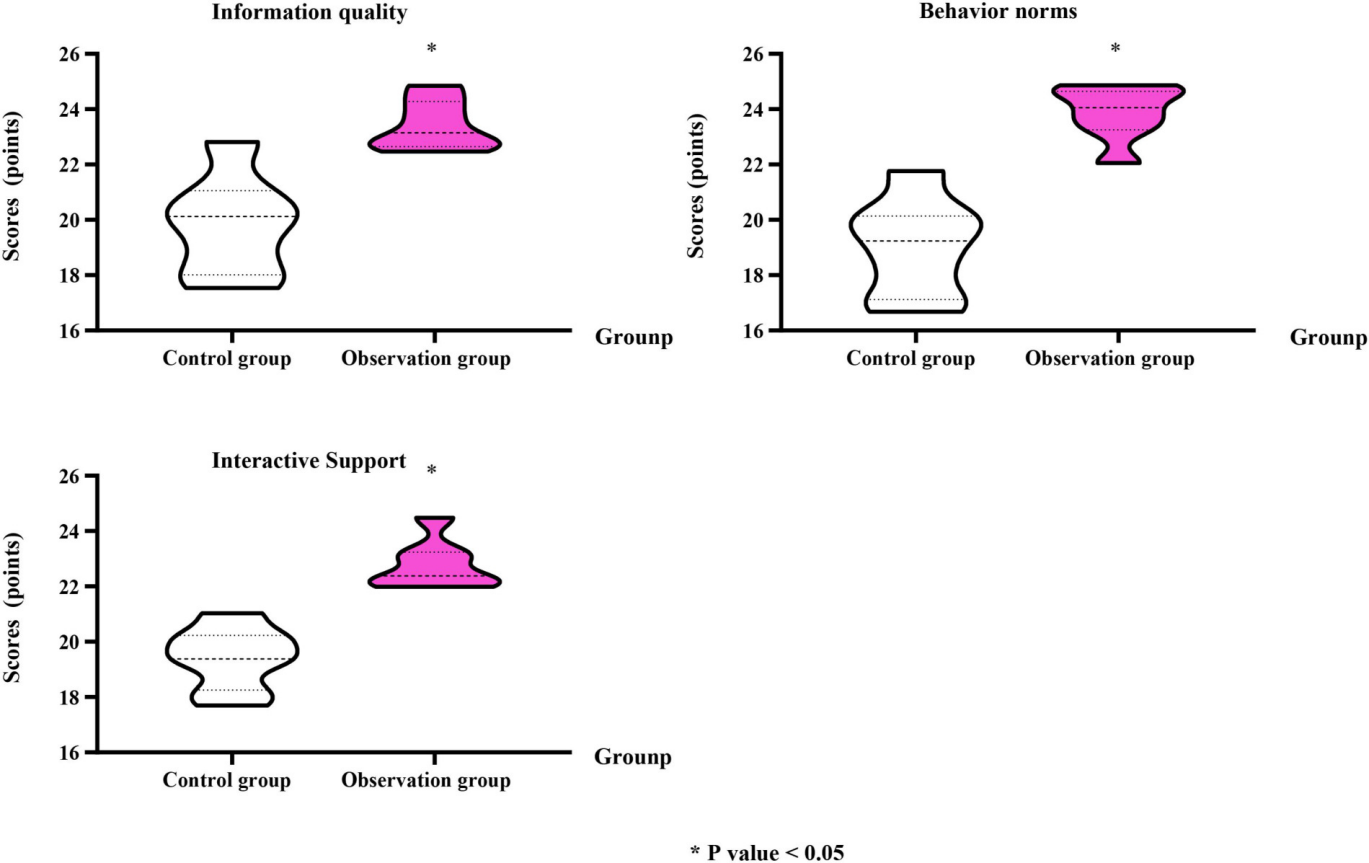
Handover quality assessed across three dimensions: information quality, behavior norms, and interactive support. Data are presented as mean ± standard deviation. *P < 0.05 indicates a statistically significant difference compared to the control group.

Compared with the CG, the OG showed significantly higher scores in information quality (OG: 23.56 ± 0.92 vs. CG: 19.96 ± 1.82; Cohen's *d* = 1.67, 95% CI: 0.86–2.46), behavior norms (OG: 23.88 ± 1.02 vs. CG: 19.04 ± 1.75; Cohen's *d* = 2.00, 95% CI: 1.11–2.86), and interactive support (OG: 22.77 ± 0.97 vs. CG: 19.42 ± 1.15; Cohen's *d* = 1.71, 95% CI: 0.89–2.51; *P* < 0.05; Figure 1).

### Mastery of the patient condition in the two groups (nurse-level)

**Figure 2:**
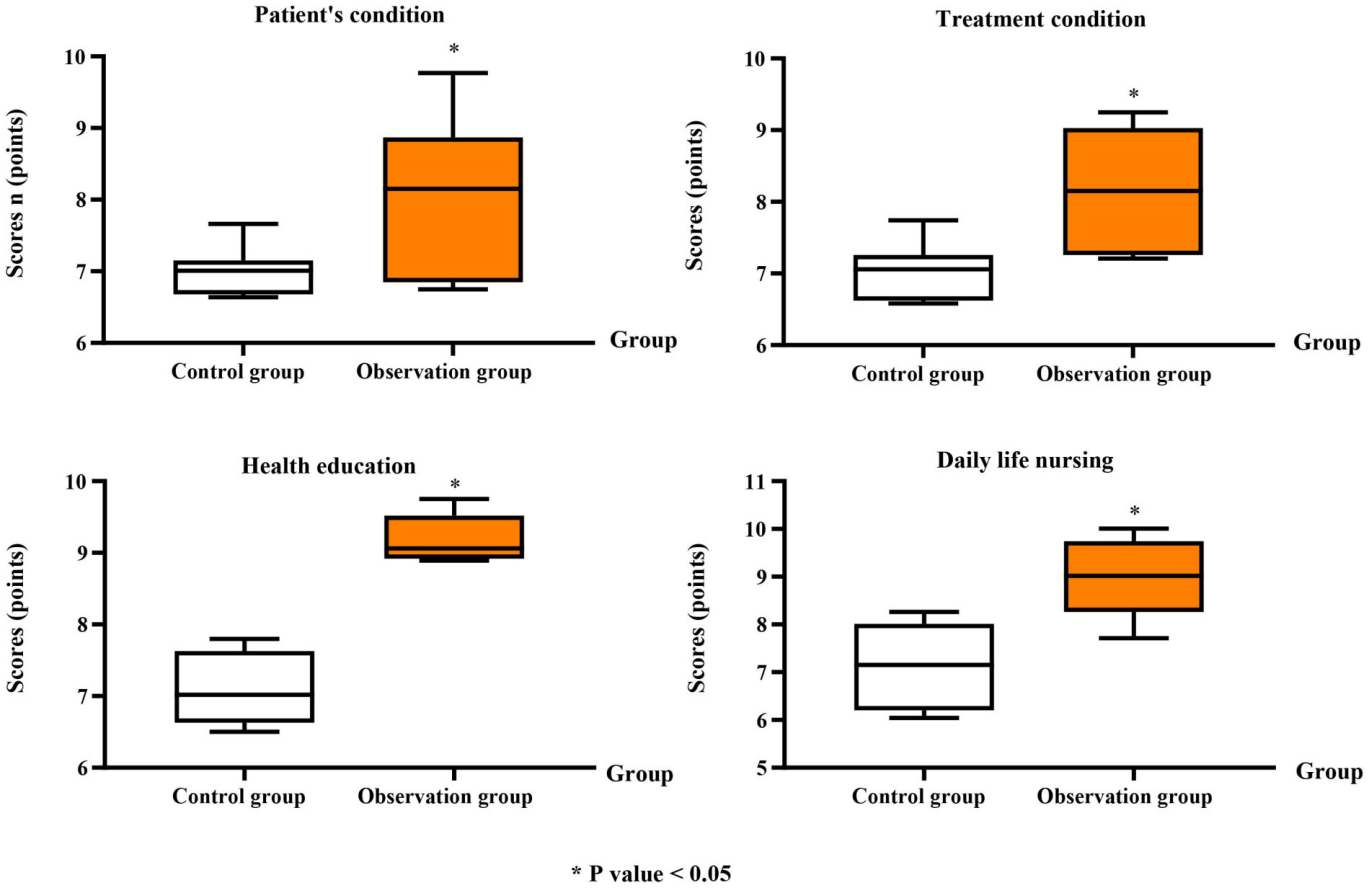
Nurses’ mastery of patient condition in the two groups. Mastery was evaluated in four areas: patient condition, treatment condition, health education, and daily life care nursing, each scored on a 10-point scale. Data are presented as mean ± standard deviation. *P < 0.05 indicates a statistically significant difference compared to the control group.

In contrast to the CG, the OG demonstrated significantly better mastery of the patient condition, including overall patient condition (OG: 7.95 ± 1.11 vs. CG: 6.87 ± 0.36; Cohen's *d* = 1.21, 95% CI: 0.40–2.01), treatment condition (OG: 8.07 ± 0.86 vs. CG: 7.01 ± 0.42; Cohen's *d* = 1.19, 95% CI: 0.38–1.99), health education (OG: 9.20 ± 0.33 vs. CG: 7.07 ± 0.52; Cohen's *d* = 1.92, 95% CI: 1.01–2.80), and daily care nursing (OG: 9.02 ± 0.86 vs. CG: 7.15 ± 0.81; Cohen's *d* = 1.44, 95% CI: 0.61–2.25; *P* < 0.05; Figure 2).

### Handover time of nurses in the two groups (nurse-level)

**Figure 3:**
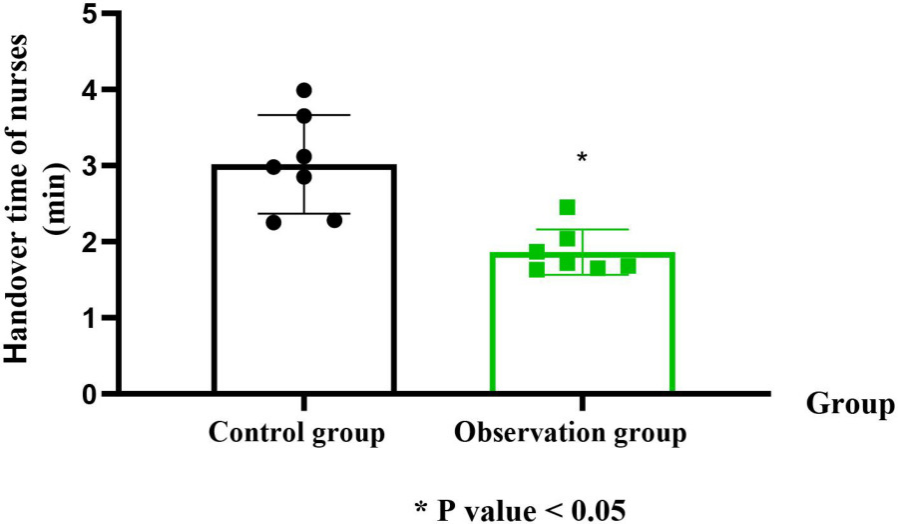
Comparison of average handover time per nurse between the two groups. Handover time was measured in minutes (min). Data are presented as mean ± standard deviation. *P < 0.05 indicates a statistically significant difference compared to the control group.

In contrast to the CG, nurses in the OG presented significantly shorter handover times (OG: 1.86 ± 0.31 min vs. CG: 3.02 ± 0.62 min; Cohen's *d* = 1.47, 95% CI: 0.64–2.28; *P* < 0.05; Figure 3).

### Defect rate of shift turnover of nurses in the two groups (nurse-level)

In contrast to the CG, the defect rate of shift turnover among nurses in the OG was lower (1/7 vs. 5/7); however, this difference was not statistically significant by two-sided Fisher's exact test (*P* = 0.103; Table 1).

**Table 1:** Defect rate of shift turnover of nurses in the two groups (unit of analysis: nurse).

Groups	*n*	Incomplete handover content	Incomplete shift record form	Defect rate
Control group	7	3	2	5 (71.43%)
Observation group	7	0	1	1 (14.29%)
*P*				0.103

*P* value is from Fisher's exact test (two-sided).

### Incidence of nursing adverse events and complaint events in the two groups (nurse-level)

The incidence of nursing adverse events was lower in the OG (1/7) than in the CG (5/7); however, this difference was not statistically significant (two-sided Fisher's exact test, *P* = 0.103). Similarly, patient complaints were fewer in the OG (0/7) than in the CG (4/7), but this difference also did not reach statistical significance (two-sided Fisher's exact test, *P* = 0.070; Table 2).

**Table 2:** Incidence of nursing adverse events and complaint events in the two groups (unit of analysis: nurse).

Groups	*n*	Nursing adverse events		Complaint events	
		Cases	%	Cases	%
Control group	7	5	71.42	4	57.14
Observation group	7	1	14.28	0	0.00
*P*		0.103		0.070	

*P* values are from Fisher's exact test (two-sided).

### Nursing satisfaction of patients in the two groups (patient-level)

Total nursing satisfaction was significantly higher in the OG (46/48, 95.83%) than in the CG (38/48, 81.25%), and this difference was statistically significant (two-sided Fisher's exact test, *P* = 0.027; Table 3).

**Table 3:** Nursing satisfaction in the two groups (unit of analysis: patient).

Groups	Cases	Very satisfied	Basically satisfied	Dissatisfied	Total satisfied
Control group	48	24	15	9	38 (81.25%)
Observation group	48	28	18	2	46 (95.83%)
*P*					0.027

Primary analysis compares total satisfaction (very + basically satisfied) vs. dissatisfied using Fisher's exact test (two-sided).

## Discussion

The nursing shift turnover process is considered a critical step in the transfer and acquisition of information among nurses (11). It is generally believed that nursing errors are often caused by incomplete or inaccurate handovers, such as omitted patient information or discontinuity of care during shift transitions, which can negatively affect follow-up nursing care and patient safety (12). Therefore, ensuring the accuracy and integrity of nursing handovers is essential for maintaining the quality and safety of patient care.

Clinically, although the traditional shift turnover process has evolved through extensive practical experience and includes a comprehensive content framework, the information to be transferred is often voluminous and poorly organized, leading to confusion, incomplete recall, and omissions (13).

As a visual and structured cognitive tool, mind mapping facilitates understanding and memory by organizing complex and unstructured information into clear visual diagrams (14). The characteristics of mind mapping include (1) accurate information delivery; (2) visualized thinking; (3) simplicity and overall clarity; (4) improved organization, optimization, and efficiency; and (5) enhanced communication and collaboration. The main advantage of mind mapping lies in its intuitive visual layout, which enables nurses to grasp key information quickly and identify problems efficiently (15).

The SBAR model was first developed and implemented by the U.S. Navy's nuclear submarine and aviation industries (16). Its purpose was to ensure efficient and standardized transmission of crucial information under high-stakes conditions, thereby reducing accidents and communication-related losses (17). In healthcare, SBAR functions as a standardized communication framework, where S (situation) refers to the patient's current clinical status, B (background) refers to relevant medical history, A (assessment) refers to the evaluation of existing problems, and R (recommendation) refers to proposed actions or risk mitigation measures. SBAR is recognized as an effective, evidence-based, and structured communication approach that ensures completeness, timeliness, accuracy, and clarity of information (18).

The results of this study indicated that, compared with the CG, the OG achieved higher scores in information quality, behavioral norms, and interactive support, while the handover time of nurses was significantly shorter. These findings suggest that combining mind mapping with the SBAR shift turnover model can effectively improve handover quality and reduce handover time during bedside shift handovers following cardiac stenting, which is consistent with previous research (19). This improvement may be attributed to the use of mind mapping as a visual cognitive tool centered around keywords and hierarchical relationships, allowing complex and scattered concepts to be presented in a structured and associative manner. Through visual elements such as lines, diagrams, and colors, mind mapping helps nurses form stronger memory links, facilitating rapid understanding and recall (20). Integrating the SBAR framework into the mind map further enhances the orderliness and logical flow of the handover process, ensuring the accuracy and completeness of information (21). In addition, the long-term use of mind mapping during shift handovers enables nurses to develop an integrated cognitive schema, allowing them to conduct handovers systematically and efficiently, ultimately improving work efficiency and shortening handover duration.

Our study also demonstrated that, compared with the CG, the OG achieved better mastery of patient condition, lower defect rates in shift turnover, fewer nursing adverse and complaint events, and higher patient satisfaction. These results indicate that combining mind mapping with the SBAR model can enhance nurses' understanding of patient condition, reduce communication errors, and improve overall care quality, findings consistent with previous studies (22). This effect may be attributed to the ability of mind mapping to promote associative thinking and critical analysis, enabling nurses to synthesize patient data, identify potential risks, and comprehensively evaluate patient needs, thereby reducing the likelihood of omissions during handover. Moreover, prior research has shown that many nursing adverse events stem from poor communication between healthcare providers, including delayed information exchange, unclear messaging, and communication barriers (23). The structured and standardized nature of the SBAR framework ensures precise, complete, and timely transmission of information, thereby minimizing errors and enhancing patient safety (24).

From a cognitive perspective, mind mapping supports dual coding and associative learning by integrating visual and verbal information, which enhances nurses' memory retention and retrieval during handover. Its clear hierarchical structure helps reduce cognitive load and facilitates rapid identification of priorities in patient care (25). Organizationally, combining mind mapping with the SBAR framework provides a shared mental model among nursing staff, promoting consistent information flow and improved teamwork efficiency (26). The visual and structured nature of this approach helps standardize communication, minimize ambiguity, and strengthen situational awareness across shifts (27). Together, these mechanisms help explain why the combined method improves both the accuracy and fluency of shift turnover.

Despite the promising results, this study has several limitations. First, the quasi-experimental design, with group allocation based on admission time rather than full randomization of patients, may introduce selection bias. Second, the single-center design and the relatively modest sample size limit the generalizability of our findings to other settings and broader populations. Future research utilizing a multicenter, randomized controlled trial with larger sample sizes is recommended to confirm the efficacy and wider applicability of the mind map-combined SBAR shift turnover model. Finally, because nurse-level and patient-level outcomes were analyzed at their respective levels without the use of multilevel modeling, residual clustering (with patients nested within nurses) may persist; future studies with larger samples should consider mixed-effects models to account for such clustering. Taken together, these findings support the feasibility and potential utility of mind map-supported SBAR for bedside handovers, while underscoring the need for confirmatory trials to establish causality.

## Conclusion

This study found that incorporating a mind map-supported SBAR structure into bedside shift handovers was associated with improvements in key process and patient-reported outcomes in a cardiology unit, including, shorter handover times, fewer observed handover defects, and higher nursing satisfaction. While the direction and magnitude of effects were consistent and clinically meaningful, the single-center setting, small nurse-level sample, and separate analysis of nurse- and patient-level endpoints limit causal inference. Accordingly, our results should be viewed as preliminary evidence of benefit rather than definitive proof of effectiveness. Future studies—ideally multicenter and randomized or cluster-randomized—are warranted to confirm effectiveness, evaluate sustainability and fidelity in routine practice, and examine downstream safety outcomes (e.g., preventable adverse events and readmissions).

## Data Availability

The raw data supporting the conclusions of this article will be made available by the authors, without undue reservation.
